# 17-hydroxiprogesterone values in healthy preterm infants

**DOI:** 10.25100/cm.v43i4.2983

**Published:** 2017-12-30

**Authors:** Víctor Clemente Mendoza-Rojas, Luis Alfonso Díaz-Martínez, Gerardo Mantilla-Mora, Gustavo Adolfo Contreras-García, Víctor Manuel Mora-Bautista, Jhon Freddy Martínez-Paredes, Alba Luz Calderón-Rojas, Carlos Augusto Gómez-Tarazona, Katherine Pinzón-Mantilla

**Affiliations:** 1 Escuela de Medicina, Facultad de Salud, Universidad Industrial de Santander, Bucaramanga, Colombia.; 2 Programa de Especialización en Pediatría, Departamento de Pediatría, Escuela de Medicina; Facultad de Salud, Universidad Industrial de Santander, Bucaramanga, Colombia.; 3 Programa de Medicina, Escuela de Medicina, Facultad de Salud, Universidad Industrial de Santander, Bucaramanga, Colombia.; 4 Programa de Microbiología y Bioanálisis, Escuela de Microbiología, Facultad de Salud, Universidad Industrial de Santander, Bucaramanga, Colombia.

**Keywords:** Neonatal screening, 17-alpha-Hidroxiprogesterone, Adrenal Hyperplasia Congenital, tamizaje neonatal, 17-hidroxiprogesterona, hiperplasia adrenal congénita

## Abstract

**Introduction:**

In preterm newborn, problems with the interpretation of 17-OHP may occur.

**Objective:**

Evaluate 17-OHP values in healthy preterm newborns until they reach the corrected gestational age.

**Methods:**

Longitudinal study of 36 preterm infants with 17-OHP evaluation using ELISA from heel blood from 3 to 5 days and thereafter every 2 weeks until the corrected gestational age. Values adjusting multiple variables such as gestational age, birth weight and sex, among others were compared. The results were analyzed against 82 healthy full-term infants.

**Results:**

In the first week of life, early term infants born within less than 34 months of gestational age show 17-OHP values that are much higher than the full term neonates. After a week, the values decrease and stabilize, but are still higher than those of full term neonates and remain so even at the corrected gestational age. (average difference of 63.0%, CI 95%: 11.8%-115.5%). 33.6% (41 samples) of a total of 122 samples taken from preterm infants were higher than 30 ng/mL.

**Conclusions:**

17-OHP values in early term infants are higher than those in full term neonates and can be related to postnatal adaptive processes. It is suggested that a second screening at the 37th week of corrected age be performed.

## Resumen

**Introducción:**

En recién nacidos pretérmino se presentan problemas para interpretar la 17-OHP.

**Objetivo:**

Evaluar los valores de 17-OHP en recién nacidos sanos pretérmino hasta cuando alcanzan el término de edad gestacional corregida.

**Métodos:**

Estudio longitudinal de 36 prematuros con evaluación de la 17-OHP por ELISA en sangre de talón desde los 3-5 días de vida y luego cada dos semanas hasta la edad gestacional de término corregida. Se comparó los valores ajustando múltiples variables como edad gestacional, peso al nacer y sexo, entre otras. Se analizaron los resultados frente a los de 82 recién nacidos a término sanos.

**Resultados:**

En la primera semana de vida, los prematuros menores de 34 semanas de edad gestacional tienen valores de 17-OHP muy superiores a los neonatos de término. Al alcanzar la semana 34 de edad gestacional corregida, los valores descienden y se mantienen estables, siempre mayores a los de término, incluso al llegar a edad a término corregida (diferencia promedio de 63.0%, IC 95%: 11.8%-115.5%). El 33.6% (41 muestras) de un total de 122 muestras hechas en los prematuros eran mayores de 30 ng/mL.

**Conclusiones:**

Los valores de 17-OHP en recién nacidos pretérmino son más altos que en neonatos a término, pudiendo ser relacionado con los procesos adaptativos postnatales. Se sugiere realizar un segundo tamizaje al llegar a la semana 37 de edad corregida.

## Introduction

Congenital adrenal hyperplasia (CAH) is an autosomal recessive disease secondary to deficiency in the synthesis of adrenal steroids, caused by a defect in the enzyme 21-hydroxylase in 90% -95% of classic form cases (OMIM: # 201910). The estimated world incidence varies between one out of every five to fifteen thousand live births ^1^ ^,^ ^2^. In its most severe form, it generates a deficiency in the production of cortisol and aldosterone, producing an adrenal crisis with high morbidity and mortality; additionally, alterations in the major sexual development (intersex) can occur in fetuses with karyotype 46XX ^2^ ^,^ ^3^.

The screening test for CAH consists of the measurement of the hormone 17-hydroxyprogesterone (17-OHP), accumulated precursor of the classic form, in a sample of dried blood on filter paper taken from the neonate's heel. Depending on the population and technique used, term infants are considered to be "abnormal" between 20 and 30 ng/mL, which means that confirmatory tests are required ^4^ ^,^ ^5^. This test presents difficulties in its application in preterm newborns because of the variability of 17-OHP values ​​due to gestational age and other factors described, such as sex, birth weight, prenatal use of corticosteroids or neonatal sepsis, among others ^3^ ^,^ ^4^ ^,^ ^6^ ^-^ ^13^. In addition, very few longitudinal studies have been carried out to determine their values ​​if they are not performed at the time when screening is recommended. Additionally, the best techniques for processing (high performance liquid chromatography - HPLC - or tandem mass spectrometry - TMS) are very expensive.

The primary objective of this work was to determine the variation of 17-OHP of healthy preterm infants until reaching the corrected gestational age. A secondary objective was to evaluate the existence of differences between the values of 17-OHP when the preterm infants arrived at the corrected term with that of full-term infants.

## Materials and Methods

A cohort study was conducted with the neonates born in the University Hospital of Santander (HUS by its acronym in Spanish) between July 2014 and August 2015. Parents of all participating children understood the study and agreed to participate, giving their written consent. The study was approved by the Ethics Committee in Scientific Research at the Universidad Industrial de Santander (Act 20 of 2013), fulfilling the Declaration of Helsinki of 1975 modified in 2004 and Resolution 8430 of 1993 of the Ministry of Health (today of Protection Social) of Colombia.

All healthy born preterm infants and two healthy term infants, born immediately after each preterm, were included in the fourteen months of the study. At the time of inclusion, all neonates had to be in normal clinical conditions for their gestational age corrected according to pediatric evaluation. It was verified that they had no perinatal history of infections, asphyxia or perinatal trauma, congenital disorders, or low birth weight according to the Fenton & Kim tables ^14^. With these same tables, the birth weight of the included patients was evaluated by means of the number of standard deviations (Z values) above or below the expected average for the sex and gestational age of the neonate.

All patients were sampled with heel prick test between the third and fifth day of life ^1^. For the preterm newborns, a clinical evaluation was performed every two weeks until completing at least 37 weeks of postconceptional age and inclusion criteria was reapplied, after which a new blood sample was taken, if applicable. The blood samples were taken with a graduated puncture device (Ascensia Microlet®) and placed on filter paper FT-2-460 to dry in open air for 12-24 hours. Later they were stored in hermetically sealed plastic bags for storage between 0°-4° C until their processing. After being processed, the remnants were frozen at -4° C to allow a prolonged conservation.

For measurement of 17-OHP levels, the Stat Fax® 2200 microplate incubator / stirrer (Awareness Technology Inc, USA) and the Chromate® 4300 micro-ELISA reader (Awareness Technology Inc, USA) were employed using the Neonatal kit 17 OH Progesterone (N-17OHP) Test System® (AccuBind ELISA Microwells, Monobind Inc., USA). The insert of this test indicates that it is a microplate ELISA with a sensitivity of 0.56 ng/mL and variation of up to 33% ^15^.

Samples with values higher than 20 ng/mL (64.52 nmol/L), the internationally accepted cut for the 17-OHP ELISA test in term neonates ^3^, were processed a second time, taking the highest of the two for analysis in order to diminish the false positives that the test itself could show due to its own variation. All children in whom an extremely high 17-OHP value was documented (>99th percentile according to gestational age and newborn weight) were offered clinical guidance and assessment by pediatric endocrinology ^6^ ^,^ ^7^ ^,^ ^12^. 

A multiple regression model was used to estimate the variation of 17-OHP according to gestational age at birth, controlling for potentially confounding variables: birth weight, sex and delivery route, gestational age, use of any prenatal corticosteroid scheme for fetal lung maturation, age at each sample intake and history of postnatal medical conditions resolved at the time of each sampling and that could cause metabolic stress, such as respiratory distress syndrome due to surfactant deficiency, hypoglycemia, jaundice or infections.

This model included a term referring to each patient used when the levels of 17-OHP found in the second and subsequent assessments are not independent ^16^; variables with a Gaussian distribution were transformed in the most appropriate way to achieve this behavior. The values are reported as median and interquartile range (IQR). Finally, the 17-OHP values of the preterm infants obtained in extra-uterine life equivalent to a gestational age corrected for term were compared with the values of the infants born at term. In all situations an α <0.05 was considered significant. The analysis was done in Stata 12.1® (Stata Corp., 2014 USA).

## Results

**Figure 1:**
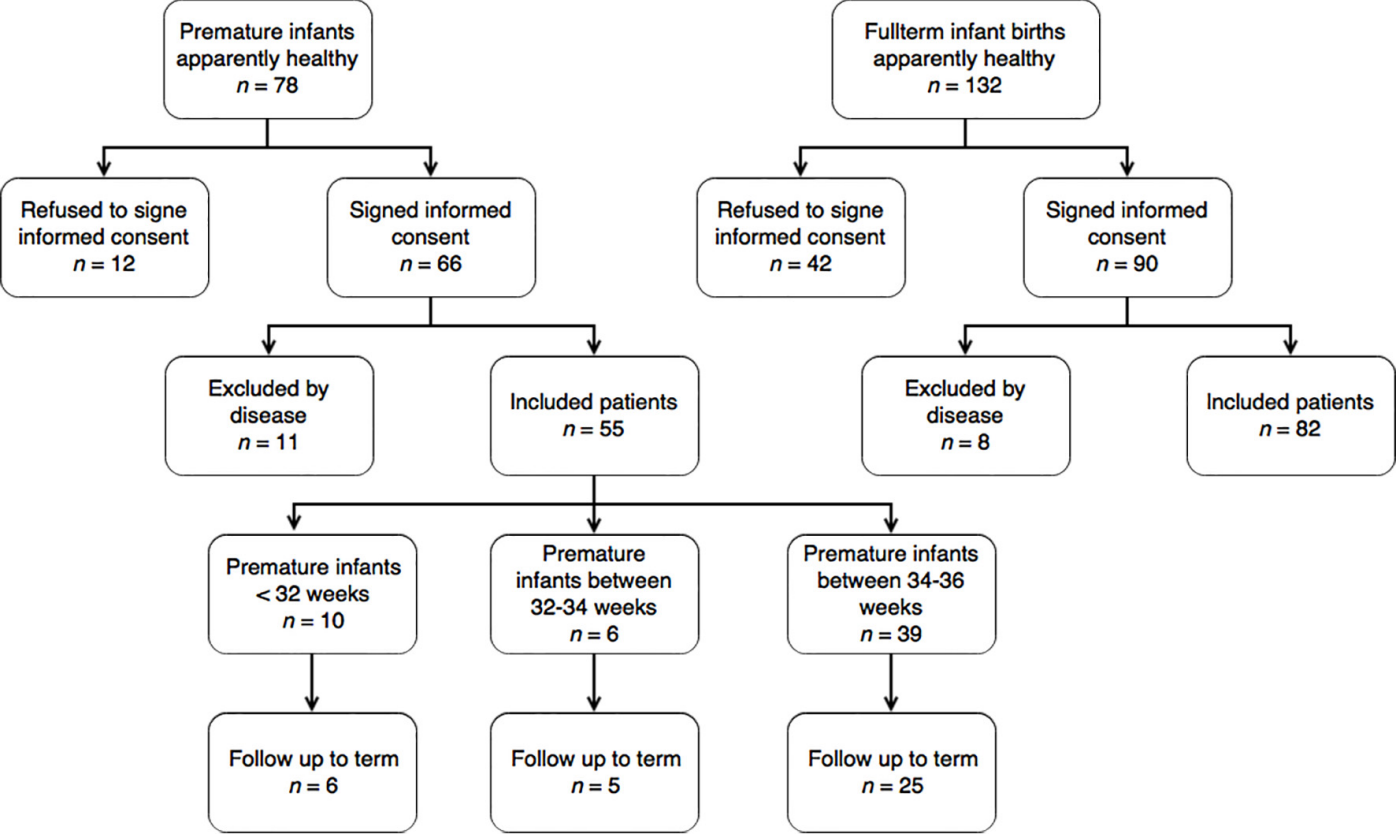
Recruitment of preterm (left) and term patients (right) flowchart.

Figure 1 presents the flowchart of uptake of preterm infants and their term counterparts. Of the 726 premature babies born in the HUS in the 14 months of recruitment, 78 were born without disease; for 66 informed consent was obtained, but 11 were not taken into account due to health problems detected later, including 55 premature babies. On the other hand, of the 132 term infants who were born healthy and who were chosen as control for the 66 premature infants in whom informed consent was obtained, 90 were authorized by their parents, but it was not possible to take into account 8 due to illness, 82 newborns were thus included. 

Table 1 shows the clinical characteristics at birth of the study's infants according to the gestational age group proposed in the two similar studies ^17^ ^,^ ^18^. In the first 17-OHP sample, five (6.1%) of the 82 neonates at term, there were 17-OHP levels above 20 ng/mL, while among the 55 premature infants, 37 (67.3%) were above this value (*p* <0.001). In this first sample, none of the term infants had levels >30 ng/mL, but 18 (32.7%) of the premature infants did. The median value of 17-OHP in term infants was 11.49 (IQR: 7.22-15.06) ng/mL, while that of the first measurement among premature infants was 25.25 (IQR: 18.05-37.47 ng/mL; *p* < 0.001).

**Table 1:** Characteristics of the study population according to gestational age at birth

Characteristic	Studied population			
	Prematures (weeks)			Term neonates (n= 82)
	<32 (n= 10)	32-33 (n= 6)	34-36 (n= 39)	
Prenatal administration of corticosteroids	9	4	21	-
Cesarean birth	10	3	24	53
Female	9	1	17	36
Patients with events that generate metabolic stress**	8	3	10	7
Jaundice	6	1	17	8
SDR	6	2	4	-
Infections	4	0	2	-
Hypoglycemia	1	1	2	2
TSH at birth (µUI/L)*	3.0 (2.2-3.7)	1.8 (1.5-3.4)	2.7 (1.7-3.6)	2.9 (1.7-4.2)
Birth weight (g)* Z score of birth weight according to gestational age*	1,470 (1,310-1,680)	2,205 (1,890- 2,380)	2,280 (2,090- 2,520)	3,230 (2,950-3,580)
	0.05 (-0.07- 0.26)	0.54 (0.10- 0.68)	-0.34 (-0.67- 0.02)	-0.28 (-0.78-0.31)

* Value as median and interquartile range.

**A patient may present more than one event that generates metabolic stress.

SDR: Respiratory distress syndrome due to pulmonary surfactant deficit.

**Figure 2:**
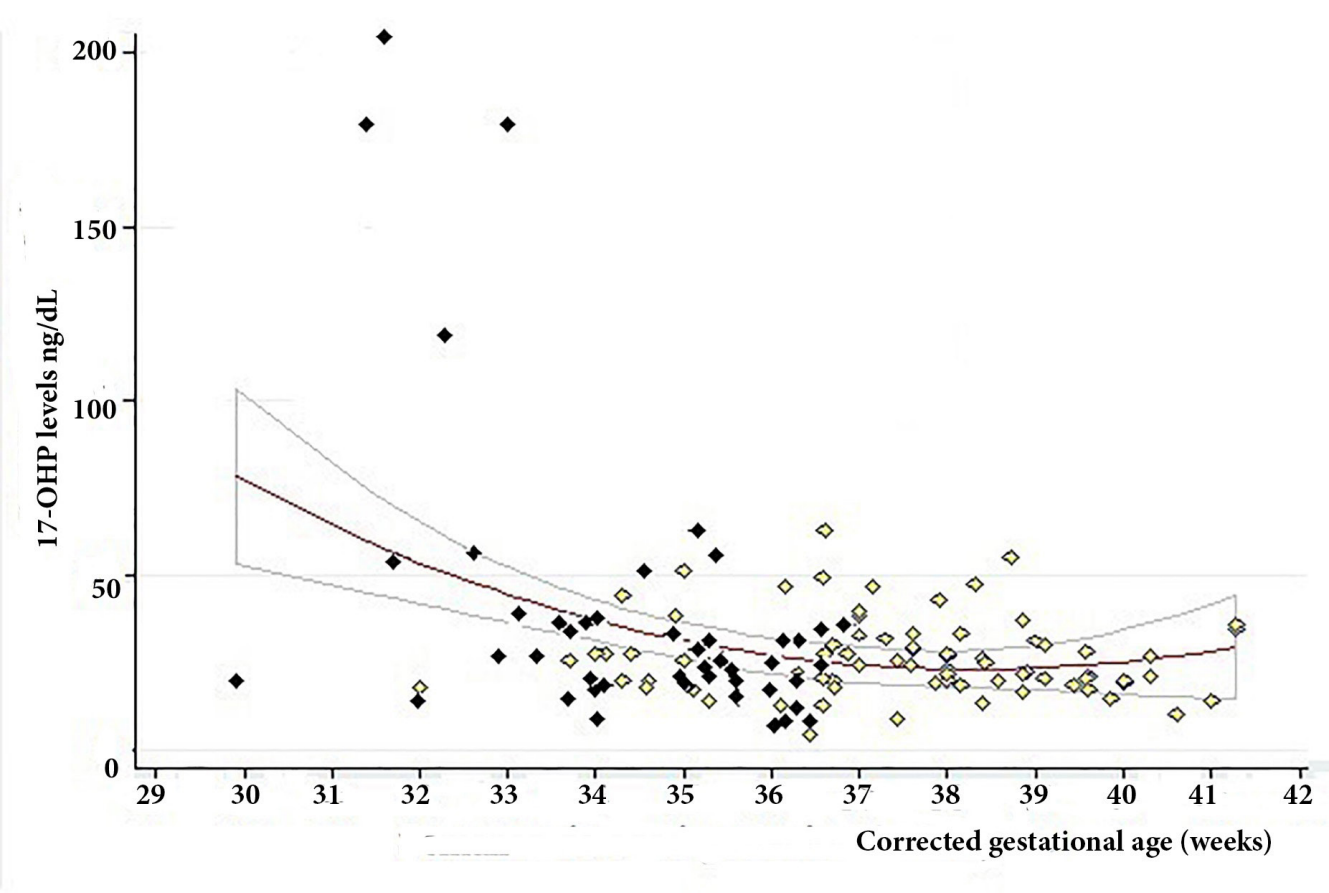
Variation of 17-OHP levels between birth and reaching the corrected gestational age. The red line is the average regression of the values of 17-OHP in the follow-up, while the gray lines its confidence band of 95%. The dark rhombuses are the values of the first shot, 3 to 5 days after birth, while the light diamonds are the later measurements during the follow-up.

Figure 2 shows the curve of 17-OHP levels variation; there is a marked reduction between weeks 28 and 32 of gestational age, to a value that remains stable until after week 34 of corrected gestational age. The parsimonious model that best explains the changes in 17-OHP levels among the neonates evaluated was of the multiple linear regression type with the 17-OHP value as the logarithm in base 10 (Table 2), where differences in the level of 17-OHP are explained only by the fact of being born preterm, regardless of the gestational age and the subsequent extra-uterine life time until the end of follow-up; the other variables included in the model (neonatal sex, birth weight, neonatal TSH and events that generate metabolic stress) modify the value of the effect of the prematurity history on the 17-OHP level but are not associated with the latter; the other variables, the prenatal use of steroids or type of delivery, neither modify the value of the effect nor are they associated by themselves. This model explains 36.8% of the variation of the logarithm of 17-OHP levels.

**Table 2:** Multiple linear regression model explains the variation of the levels of the 17 OHP.

Variable	β	CI 95%	*p*-value
Prematurity	0.344	0.222 a - 0.467	<0.001
Female	0.024	-0.055 -a 0.103	0.552
Birth weight (each 100 g)	-0.003	-0.011 -a 0.006	0.548
Neonatal TSH	-0.004	-0.021 -a 0.013	0.629
Conditions producing metabolic stress	-0.075	-0.187 -a 0.037	0.188

The median 17-OHP among premature infants when they reached the end of corrected gestational age was 21.64 (IQR: 18.82 to 31.30) ng/mL; none of the patients with 17-OHP> 20 ng/mL (positive for HAC screening) was later confirmed as a carrier of the disease.

## Discussion

In the present study, the behavior of measured 17-OHP values from heel prick test in healthy preterm infants showed a tendency to decrease progressively from week 29 of gestational age to a homogeneous value after 33-34 weeks, reaching a stable value at 37 weeks regardless of the initial gestational age. However, the value of 17-OHP in preterm infants remained high, even when they reach the corrected term of gestation.

In the test for CAH, prematurity causes the standard threshold for term infants (≥20 ng/mL) not to be a proper value when screening is done: in infants less than 1,500 g weight one high number presented false positives, as reported by Ryckman *et al* ^10^. From The results of this study, it may be concluded that the increased false positives is so because the threshold value should be greater, even when they reach the age of corrected term. Hence, it is tempting to propose a higher threshold between 30 and 50 ng/mL, a figure that should be validated by studies to establish the diagnostic yield of this proposed value, including an adequate number of patients for such studies. With a cut-off point of 30 ng/mL in our population, the false positive rate in term neonates is null, compared to the cut-off of 20 ng/mL, which is 9.0%. This is not the first time that raising this threshold has been proposed: Cavarzere *et al*. ^11^, as proposed in 22 ng/mL (70 nmol/L) when preterm reach 36 weeks post-conception, or Nordenström *et al*. ^19^, who consider a value of 46 ng/mL (150 nmol/L) .

Our data are similar to those reported by Al Saedi *et al*. ^17^, who were the first to describe weekly 17-OHP values in preterm infants up to 37 weeks. On the other hand, the values obtained in this investigation are not as high as those reported by Linder *et al*. ^18^, who included sick preterm infants. These two studies and the present are the only longitudinal studies available to date.

Multiple factors have been described that cause high levels of 17-OHP in preterm infants, such as maternal conditions, environmental influences, infections and respiratory distress mainly and, in general, the stress generated by complications and treatments derived from prematurity. Other reasons seem to be the immaturity of the liver function to degrade 17-OHP, higher production of adrenal steroids from the fetal remnant, the immaturity of the hypothalamic pituitary axis, immaturity of the enzymes in the glucocorticoid pathway to cross-reactions with polar steroids; any reflection on the adaptive demands that being born premature makes an immature fetus ^20^. The data of the present study showed that the only factor that conditions significant variation of 17-OHP is the gestational age at which it is born, especially in children under 34 weeks. No dependence was found on the other variables analyzed, such as sex and birth weight, a situation similar to that found by Linder *et al*. ^17^, but differing with that described by Ballerini *et al* ^13^.

All the above explains the existence of different criteria in determining the age at which to perform screening in preterm infants ^12^, as well as the suggestion to repeat the sample every two weeks or as Huet *et al*. ^22^, not to perform screening in preterm infants <32 weeks given the limitations involved in interpreting the results.

When evaluating the influence of postnatal life, it was observed that the value of 17-OHP in preterm infants when they reach 37 corrected weeks, is greater than the term neonates. It is important to take into account that the values ​​of 17-OHP in our term neonates coincide with that reported in the literature ^6^ ^,^ ^22^ ^,^ ^23^. This suggests that the immaturity of the adrenal cortex is more noticeable when it is more severe is the prematurity, subsequently stabilizing and allowing a return to a 17-OHP value close to that determined by the biological programming of the adrenal, which is observable in term neonates. The complete functioning of the adrenal axis is not fully understood, but the fetal area of ​​the adrenal gland persists and seems not to be affected by postnatal age ^20^ ^,^ ^23^. The greater activity of the adrenal cortex in healthy preterm is part of its adaptive process, and it is expected that the cut-off value of hyper-17-hydroxyprogesteronemia would be higher in these cases ^21^ ^,^ ^24^ ^,^ ^25^.

On the other hand, Cavarzere *et al*. ^11^, mention that there are cases of hyper-17-OHPnemia in preterm neonates, of a physiological nature, levels that remain up to 4-6 months of postnatal life, which would make it even more difficult to interpret the screening values. In our study group, no such case was evidenced.

Limitations of this study are related to losses in follow-up, both due to socioeconomic reasons that prevented families from attending the controls, which particularly impacted the follow-up of infants of younger gestational age, in whom it is very common that there were conditions of illness that forced us to exclude them from the analysis. However, the linear regression model used is robust and allows the inclusion of patients without the full desirable follow-up.

We hope that this information may serve as the basis for implementing universal screening in Colombia, although it has already been promoted by the Government as a recommendation since 2013 ^26^. The most important limitation for this development is the cost of the tests to measure 17-OHP, because technologies such as UM-ELISA or HPLC (tandem mass spectrometry) are difficult to acquire in the country and are even more expensive when measuring a single analytic per test.

## Conclusion

The behavior of 17-OHP values taken from heel prick test in healthy preterm infants presented a tendency to decrease progressively from 30 to 34 weeks of postconceptional age, being similar from this point and up to 37 weeks, regardless of the gestational age of birth. Therefore, we suggest performing screening in preterm infants starting at 34 weeks and repeating at 37 corrected weeks.

The 17-OHP values of heel blood in preterm infants when they reach 37 weeks are higher than in full-term infants, and is inversely correlated with gestational age and not with the use of prenatal corticosteroids, sex, birth weight nor birth path.

The results of the present study suggest the possibility of establishing only one cut-off value for 17-OHP taken from heel prick test , for all preterm neonates after reaching 37 weeks of postconceptional age.
