# Supplementary material for: 17-hydroxiprogesterone values in healthy preterm infants
Source: Colomb Med (Cali). 2017 Dec 30;48(4):161–6. doi: 10.25100/cm.v43i4.2983 (PMC5896722; doi:10.25100/cm.v43i4.2983)
Supplement: Related texts

# Research article: Valores de 17-hidroxiprogesterona en recién nacidos prematuros sanos

## Introducción

La hiperplasia adrenal congénita (HAC) es una enfermedad autosómica recesiva secundaria a la deficiencia en la síntesis de esteroides adrenales, causada por un defecto en la enzima 21-hidroxilasa en el 90%-95% de los casos forma clásica (OMIM: #201910). La incidencia mundial estimada varía entre uno para cada cinco a quince mil nacidos vivos ^1^^,^^2^. En su forma más severa, genera deficiencia en la producción de cortisol y aldosterona, produciendo crisis adrenal con una alta morbimortalidad; adicionalmente, pueden presentarse alteraciones en el desarrollo sexual mayor (intersexo) en fetos con cariotipo 46XX ^2^^,^^3^. 

La prueba de tamizaje para la HAC consiste en la medición de la hormona 17- hidroxiprogesterona (17-OHP), precursor acumulado de la forma clásica, en una muestra de sangre seca en papel filtro tomada del talón del neonato; dependiendo de la población y de la técnica empleada, se considera para los neonatos de término como "anormal" un valor de entre 20 o 30 ng/dL, lo que obliga a realizar las pruebas confirmatorias ^4^^,^^5^. Esta prueba tiene dificultades para su aplicación en los recién nacidos pretérminos por la variabilidad de los valores de la 17-OHP por la edad gestacional y otros factores descritos, como sexo, peso al nacer, uso prenatal de corticoides o sepsis neonatal, entre otros ^3^^,^^4^^,^^6^^-^^13^. Además, muy pocos estudios longitudinales se han realizado para determinar sus valores en caso de no realizarse en el momento en el que se recomienda hacer el tamizaje. Adicionalmente las mejores técnicas para su procesamiento (cromatografía líquida de alto rendimiento - HPLC en inglés, espectrometría de masas en tándem - TMS) son de alto costo.

El objetivo primario de este trabajo fue determinar la variación de la 17-OHP de neonatos pretérmino sanos hasta llegar a la edad estacional corregida. Como objetivo secundario se tuvo evaluar la existencia de diferencias entre los valores de 17-OHP cuando los prematuros llegan al término corregido con el de los neonatos nacidos a término.

## Materiales y Métodos

Se realizó un estudio de cohorte con los neonatos que nacieron en el Hospital Universitario de Santander (HUS) entre julio de 2014 y agosto de 2015. Los padres de todos los niños participantes entendieron el estudio y aceptaron participar, dando su consentimiento informado. Este estudio fue aprobado por el Comité de Ética en Investigación Científica de la Universidad Industrial de Santander (Acta 20 de 2013), cumpliendo la Declaración de Helsinki de 1975 modificada en 2004 y la Resolución 8430 de 1993 del Ministerio de Salud (hoy de la Protección Social) de Colombia.

Se captaron todos los neonatos pretérminos nacidos sanos y los dos neonatos de término sanos que nacieron inmediatamente después de cada prematuro incluido en los catorce meses del estudio. Al momento de ser incluidos, todos los neonatos debían estar en condiciones clínicas de normalidad para su edad gestacional corregida según valoración pediátrica. Se verificó que no tuviesen historial perinatal de infecciones, asfixia o trauma perinatal, trastornos congénitos, ni bajo peso al nacer según las tablas de Fenton^14^); con estas mismas tablas, el peso al nacer de los pacientes incluídos, fue evaluado por medio del número de desviaciones estándar (valores Z) por arriba o abajo del promedio esperado para el sexo y la edad gestacional del neonato. 

A todos los pacientes se les tomaron muestras de sangre total del talón entre el tercer y quinto día de vida ^1^. Para los recién nacidos prematuros se realizó una evaluación clínica cada dos semanas hasta completar al menos 37 semanas de edad postconcepcional y se reaplicaron los criterios de inclusión, luego de lo cual se procedía a tomar una nueva muestra de sangre, si aplicaba. Las muestras de sangre se tomaron con un dispositivo de punción graduada (Ascensia Microlet^®)^ y se colocaron en papel filtro FT-2-460 para dejarlas en secado al aire libre por 12-24 horas. Posteriormente se guardaron en bolsas plásticas de sellado hermético para almacenamiento entre 0° y 4° C hasta su procesamiento. Luego de ser procesadas, los remanentes se congelaron a -4° C para permitir una conservación prolongada. 

Para la medición de los niveles de 17-OHP se usó el incubador/agitador de microplacas Stat Fax^®^ 2200 (Awareness Technology Inc, USA) y el lector de micro-ELISA Chromate^®^ 4300 (Awareness Technology Inc, USA) utilizando el kit Neonatal 17 OH Progesterona (N-17OHP) Test System^®^ (AccuBind ELISA Microwells, Monobind Inc, USA). El inserto de esta prueba indica que es un ELISA de microplaca con una sensibilidad de 0.56 ng/mL y variación del hasta el 33% ^15^. 

Las muestras con valores superiores a 20 ng/mL (64.52 nmol/L), el corte aceptado internacionalmente para la prueba de ELISA 17-OHP en neonatos a término ^3^, se procesaron por segunda vez, tomándose para el análisis el más alto de las dos mediciones resultantes, buscando disminuir los falsos positivos que pudiese arrojar la prueba por su propia variación. A todos los niños en quienes se documentó un valor de 17-OHP extremadamente alto (>percentil 99 según la edad gestacional y el peso del recién nacido) se les ofreció orientación clínica y valoración por endocrinología pediátrica ^6^^,^^7^^,^^12^. 

Se utilizó un modelo de regresión múltiple para estimar la variación de la 17-OHP según la edad gestacional al nacer controlando por las potenciales variables de confusión: peso al nacer, sexo y vía del parto, edad gestacional, uso de cualquier esquema de corticoesteroides prenatales para maduración pulmonar fetal, edad a la toma de cada muestra e historial de condiciones médicas postnatales resueltas al momento de cada toma de muestra y que pudiesen causar estrés metabólico, como síndrome de dificultad respiratoria por déficit de surfactante, hipoglucemia, ictericia o infecciones. 

Éste modelo incluyó un término referido a cada paciente en la medida que los niveles de 17-OHP hallados en las segundas y subsiguientes valoraciones no son independientes^16^; las variables con distribución distinta a la gaussiana se trasformaron a la forma más adecuada para lograr este comportamiento. Los valores se reportan como mediana y recorrido intercuartil (RIQ). Finalmente, los valores de 17-OHP de los neonatos prematuros obtenidos en la vida extrauterina equivalente a una edad gestacional corregida de término se compararon con los valores de los neonatos nacidos a término. En todas las situaciones se consideró significativa un α <0.05. El análisis se hizo en Stata 12.1^®^ (Stata Corp., 2014 USA). 

## Resultados

La Figura 1 presenta el flujograma de captación de los recién nacidos prematuros y sus contrapartes de término. De los 726 prematuros que nacieron en el HUS en los 14 meses de captación, 78 nacieron sin enfermedad; para 66 se obtuvo consentimiento informado, pero 11 no fueron tenidos en cuenta por problemas de salud detectados luego, incluyéndose así 55 prematuros. Por otro lado, de los 132 neonatos de término que nacieron sanos y que se escogieron como control de los 66 prematuros en quienes se obtuvo consentimiento informado, 90 fueron autorizados por sus padres, pero no fue posible tener en cuenta 8 por enfermedad, incluyéndose finalmente 82 neonatos. 

**Figura 1:** Flujograma de captación de pacientes prematuros (izquierda) y de término (derecha).

En la Tabla 1 se presentan las características clínicas al nacer de los neonatos del estudio según la agrupación de edad gestacional propuesta en los dos estudios similares ^17^^,^^18^. En la primera muestra de 17-OHP, cinco (6.1%) de los 82 neonatos de término presentaba niveles de 17-OHP por arriba de 20 ng/mL mientras que entre los 55 prematuros, 37 (67.3%) estaban por arriba de este valor (*p* <0.001). En esta primera muestra, ninguno de neonatos de término presentaba niveles >30 ng/mL, pero si 18 (32.7%) de los prematuros. La mediana del valor de 17-OHP en los neonatos de término fue de 11.49 (RIQ: 7.22-15.06) ng/mL, mientras que la de la primera medición entre los prematuros fue de 25.25 (RIQ: 18.05).

**Tabla 1:** Características de la población del estudio según edad gestacional al nacer.

Característica	Población estudiada			
	Prematuros (semanas)			Neonatos de término (n= 82)
	<32 (n= 10)	32-33 (n= 6)	34-36 (n= 39)	
Administración prenatal de corticosteroides	9	4	21	-
Parto por cesárea	10	3	24	53
Sexo femenino	9	1	17	36
Pacientes con eventos que generan estrés metabólico**	8	3	10	7
*Ictericia*	6	1	17	8
*SDR*	6	2	4	-
*Infecciones*	4	0	2	-
*Hipoglucemia*	1	1	2	2
TSH al nacer (µUI/L)*	3.0 (2.2-3.7)	1.8 (1.5-3.4)	2.7 (1.7-3.6)	2.9 (1.7-4.2)
Peso al nacer (g)*	1,470 (1,310-1,680)	2,205 (1,890-2,380)	2,280 (2,090-2,520)	3,230 (2,950-3,580)
Puntaje Z del peso al nacer según la edad gestacional*	0.05 (-0.07-0.26)	0.54 (0.10-0.68)	-0.34 (-0.67-0.02)	-0.28 (-0.78-0.31)

* Valor como mediana y recorrido intercuartil

** Un paciente puede presentar más de un evento que generase estrés metabólico

SDR: Síndrome de dificultad respiratoria por déficit de surfactante

En la Figura 2 se aprecia la curva de variación de los niveles de 17-OHP; hay una reducción acentuada entre las semanas 28 y 32 de edad gestacional, a un valor que permanece estable a partir de la semana 34 de edad gestacional corregida. El modelo parsimonioso que explica mejor los cambios en los niveles de 17-OHP entre los neonatos evaluados fue de tipo regresión lineal múltiple con el valor de 17-OHP como logaritmo en base 10 (Tabla 2), en donde las diferencias en el nivel de 17-OHP se explican sólo por el hecho de nacer pretérmino, independientemente de la edad gestacional y el subsecuente tiempo de vida extrauterina hasta llegar al término; las demás variables incluidas en el modelo (sexo neonatal, peso al nacer, TSH neonatal y eventos que generan estrés metabólico) modifican el valor del efecto del historial de prematuridad sobre el nivel de 17-OHP pero no están asociadas con este último; las demás variables, el uso prenatal de esteroides o la vía del parto, ni modifican el valor del efecto ni están asociadas por sí mismas. Este modelo explica el 36.8% de la variación del logaritmo de los niveles de 17-OHP. 

**Figura 2:** Variación de los niveles de 17-OHP entre el nacimiento y el llegar al término de edad gestacional corregida. La línea roja es la regresión promedio de los valores de 17-OHP en el seguimiento, mientras que las líneas grises su banda de confianza del 95%. Los rombos oscuros son los valores de la primera toma, 3 a 5 días luego del nacimiento, mientras que los rombos claros son las mediciones posteriores durante el seguimiento.

**Tabla 2:** Modelo de regresión lineal múltiple que mejor explica la variación de los niveles del 17 OHP

Variable	β	IC 95%	Valor de *p*
Prematuridad	0.344	0.222-0.467	<0.001
Sexo femenino	0.024	-0.055-0.103	0.552
Peso al nacer (cada 100 g)	-0.003	-0.011-0.006	0.548
TSH neonatal	-0.004	-0.021-0.013	0.629
Condiciones que producen estrés metabólico	-0.075	-0.187-0.037	0.188

 La mediana de la 17-OHP entre los prematuros cuando llegaron al término de la edad gestacional corregida fue de 21.64 (RIQ: 18.82-31.30) ng/mL; ninguno de los pacientes con 17-OHP >20 ng/mL (positivos para tamizaje de HAC) fue confirmado luego como portador de la enfermedad. 

## Discusión

En el presente estudio, el comportamiento de los valores del 17-OHP medidos del talón en los recién nacidos pretérmino sanos presentó una tendencia a disminuir de forma progresiva desde la semana 29 de edad gestacional hasta un valor homogéneo a partir de las 33-34 semanas, alcanzando en las 37 semanas un valor estable sin importar la edad gestacional inicial. Sin embargo, el valor de la 17-OHP en neonatos prematuros permaneció elevado, aun cuando llegan al término corregido de la gestación.

En la prueba para HAC, la prematuridad hace que el umbral estándar para recién nacidos a término (≥20 ng/mL) no sea un valor adecuado cuando se hace tamizaje: en los neonatos con peso inferior a 1,500 g se presenta un alta número de falsos positivos, como lo reportado por Ryckman *et al*^10^. Con los resultados del presente estudio es posible concluir que este mayor volumen de falsos positivos entre prematuros está dado porque el valor umbral debería ser superior, incluso cuando alcanzan la edad de término corregida. De allí que sea tentador proponer un umbral mayor, entre 30 y 50 ng/mL, cifra que habría que validar con estudios para establecer cuál es el rendimiento diagnóstico de este valor propuesto, incluyendo para tales estudios un número adecuado de pacientes. Con un punto de corte de 30 ng/mL en nuestra población, la tasa de falsos positivos en neonatos a término es nula, en comparación con el corte de 20 ng/mL que es del 9.0%. No es la primera vez que se propone elevar este umbral: Cavarzere *et al*. ^11^, lo proponen en 22 ng/mL (70 nmol/L) cuando los prematuros alcanzan las 36 semanas de edad postconcepcional, o Nordenström *et al*. ^19^, quienes consideran un valor de 46 ng/mL (150 nmol/L). 

Nuestros datos son similares a los reportados por Al Saedi *et al*. ^17^, quienes fueron los primeros en describir semanalmente los valores de 17-OHP en neonatos pretérmino hasta llegar a 37 semanas. Por otra parte, los valores obtenidos en esta investigación no son tan altos como los reportados por Linder *et al*. ^(^^18^, quiénes incluyeron prematuros enfermos. Estos dos estudios y el presente son los únicos longitudinales disponibles a la fecha. 

Se han descrito múltiples factores causantes de los valores elevados de 17-OHP en neonatos pretérmino, como las condiciones maternas, influencia del medio ambiente, infecciones y dificultad respiratoria principalmente y, en general, el estrés generado por las complicaciones y tratamientos derivados de la prematuridad. Otras razones aparentemente son la inmadurez de la función hepática para degradar la 17-OHP, producción mayor de esteroides adrenales del remanente fetal, la inmadurez del eje hipotálamo hipofisario, inmadurez de las enzimas en la vía de los glucocorticoides hasta reacciones cruzadas con los esteroides polares; todo reflejo de las demandas adaptativas que el nacer prematuro hace a un feto inmaduro^20^. Los datos del presente estudio mostraron que el único factor que condiciona variación significativa de la 17-OHP es la edad gestacional a la que se nace, especialmente en menores a 34 semanas. No se halló dependencia de las otras variables analizadas, como sexo y peso al nacer, situación similar a la encontrada por Linder *et al*. ^17^, pero difiriendo con lo descrito por Ballerini *et al* ^13^.

Todo lo anterior explica la existencia de diferentes criterios para determinar la edad en la cual realizar el tamizaje en neonatos pretérmino ^12^, así como también la sugerencia de repetir cada dos semanas la muestra o como la de Huet *et al*. ^22^, de no realizar tamizaje en neonatos pretérmino <32 semanas dadas las limitaciones para interpretar el resultado. 

Al evaluar la influencia de la vida posnatal, se observó que el valor de 17-OHP en neonatos pretérmino cuando llegan a las 37 semanas corregidas, son mayores que los neonatos a término. Es importante tener en cuenta, que los valores de 17-OHP en nuestros neonatos a término coinciden con lo reportado en la literatura ^6^^,^^22^^,^^23^. Ello permite sugerir que la inmadurez de la corteza adrenal es más notoria cuando es más severa es la prematurez, estabilizándose posteriormente y permitiendo retomar un valor de la 17-OHP cercano al que determina la programación biológica de la adrenal, que es el observable en neonatos a término. El funcionamiento completo del eje adrenal no está del todo dilucidado, pero la zona fetal de la glándula adrenal persiste y parece no afectarse por la edad posnatal ^20^^,^^23^. La mayor actividad de la corteza adrenal en pretérminos sanos es parte de su proceso adaptativo, siendo esperable que el valor de corte de la 17-OHP fuese mayor en estos casos ^21^^,^^24^^,^^25^.

Por otro lado, Cavarzere *et al*. ^11^, mencionan que existen casos de híper 17-OHPnemia en neonatos pretérmino, de naturaleza fisiológica, niveles que permanecen hasta 4-6 meses de vida posnatal, lo que dificultaría aún más la interpretación de los valores de tamizaje. En nuestro grupo de estudio no se evidenció caso alguno.

Las limitaciones del presente trabajo están relacionadas con las pérdidas en el seguimiento, tanto por razones socioeconómicos que impedían a las familias asistir a los controles, lo que es en particular impactan el seguimiento de los neonatos de menor edad gestacional, en quienes es muy frecuente que existiesen condiciones de enfermedad que obliga a excluirlos del análisis. Sin embargo, el modelo de regresión lineal empleado es robusto y permite incluir pacientes sin el seguimiento completo deseable.

Esperamos que esta información pueda servir de base para implementar del tamizaje universal en Colombia, aunque ya está promovido gubernamentalmente como una recomendación desde 2013 ^26^. La limitación más importante para este desarrollo es el costo de las pruebas para medir 17-OHP, debido a que tecnologías como UM-ELISA o HPLC (espectrometría de masas en tándem) son de difícil adquisición en el país y son aún más costosas cuando se realiza la medición de un único analito por prueba.

## Conclusiones

El comportamiento de los valores del 17-OHP hechos del talón en los recién nacidos pretérmino sanos presentó una tendencia a disminuir progresivamente desde las 30 a las 34 semanas de edad postconcepcional, siendo similar a partir de este punto y hasta 37 semanas, independientemente de la edad gestacional de nacimiento. Por ello, sugerimos realizar en tamizaje en los neonatos pretérmino a partir de las 34 semanas y repetirlo a las 37 semanas corregidas.

Los valores de 17-OHP de sangre de talón en neonatos prematuros cuando llegan a las 37 semanas son mayores que en los recién nacidos a término, y está correlacionado inversamente con la edad gestacional y no con uso de corticoides prenatales, sexo, peso al nacer ni vía del parto. 

Los resultados del presente estudio sugieren la posibilidad de establecer sólo un valor de corte para la 17-OHP en sangre del talón, para todos los neonatos pretérmino luego de alcanzar las 37 semanas de edad postconcepcional.
